# Barriers and facilitators of collaboration during the implementation of vocational rehabilitation interventions: a systematic review

**DOI:** 10.1186/s12888-024-06223-y

**Published:** 2024-11-01

**Authors:** Yvonne Noteboom, Alexandra W. A. Montanus, Femke van Nassau, George Burchell, Johannes R. Anema, Maaike A. Huysmans

**Affiliations:** 1 Public and Occupational Health, Amsterdam University Medical Centers (UMC), Van Der Boechorststraat 7, Amsterdam, 1081 BT the Netherlands; 2 Amsterdam Public Health (APH), Societal Participation and Health, Amsterdam UMC, Amsterdam, the Netherlands; 3 VU University Library, De Boelelaan 1117, Amsterdam, 0F-039 - 1081 HV the Netherlands

**Keywords:** Mental health problems, Mental health care sector, Social security sector, Collaboration, Vocational, Rehabilitation, Return to work, Implementation, Qualitative, Systematic review

## Abstract

**Background:**

Stakeholders from the mental health care sector and the social security sector are often involved in the implementation of vocational rehabilitation (VR) interventions, so-called coordinated or integrated program, as clients need support from both fields. Collaboration of the involved stakeholders from both sectors is therefore important. In this study, a review was performed to provide an overview of the barriers and facilitators for collaboration during the implementation of coordinated or integrated vocational rehabilitation interventions.

**Methods:**

A systematic review (PROSPERO ID CRD42023404823) was performed in the databases of Medline PubMed (*n* = 11.511), Web of Science (*n* = 4821), and PSYCINFO (*n* = 368). We used the AI-driven tool ASReview to support the screening process, conducted by two researchers independently. A thematic content analysis was performed to analyse the reported barriers and facilitators. Appraisal of the quality of included studies was conducted using Critical Appraisal Skills Programme (CASP).

**Results:**

We included 105 of the 11,873 identified articles for full text screening, of which 26 were included for final analysis. Six themes of barriers and facilitators were found: attitude and beliefs, engagement and trust, governance and structure, practical issues, professionals involved, and client-centeredness. We found a reporting quality between 8 and 20, based on CASP.

**Conclusion:**

We found that a positive attitude towards and belief of those involved in collaboration during coordinated of integrated VR interventions can enhance collaboration. Moreover, a negative attitude or lack of trust, most often found among mental health professionals, hindered collaboration. Collaboration between stakeholders from different sectors could be increased by improving positive attitudes and mutual trust and increasing knowledge about each other’s expertise. Also sharing success stories, co-location of professionals, and having a clear governance were found to be a factor in collaborations’ success.

**Supplementary Information:**

The online version contains supplementary material available at 10.1186/s12888-024-06223-y.

## Introduction

People with mild and severe mental health problems are more often unemployed than people without mental health problems [1, 2]. At the same time, having a job is good for mental health [3, 4]. People who work (under good working conditions) experience higher self-esteem, have increased (self-rated) personal health and social lives, and have higher incomes [1, 3, 5, 6]. Moreover, higher rates of employment among people with mental health problems positively affect society: more participation in paid work saves on health care costs, decreases the payment of social benefits, and increases the participation of a larger potential working force [1]. Thus, both people with mental health problems and society on the whole can benefit from the increased work participation of this population. It is therefore important to overcome the barriers that people with mental health problems still experience in gaining or returning to work, such as stigma, lack of work experience or skills, and lack of self-esteem and trust [7–10].

To overcome these barriers, vocational rehabilitation (VR) programs are developed to support people in gaining employment or returning to work. Waddel et al. define these programs as *“Whatever helps someone with a (physical or mental) health problem to stay at, return to and remain in work: it is an idea and an approach as much as an intervention or a service.” *[11] In practice, vocational rehabilitation for people with severe mental health problems is mostly offered through targeted interventions [12, 13]. Such targeted interventions are comprised of different approaches, often focusing on mental health-related well-being and skills or on work-related skills. Examples of mental health related interventions are cognitive therapy and stress management. Examples of work-related interventions are workshops, job interview training classes, counselling, and sheltered workplaces [12, 13]. Mental health care providers are often the main stakeholders responsible for the delivery of interventions focusing on mental health while social work companies or social security institutes are more often responsible for providing work-related interventions. The social security sector is also involved in supporting the unemployed by providing and counselling on social benefits [14–19].

The separate (rather than integrated) approach to these interventions thus comes from the different sectors that care providers are operating within [14–19]. Traditionally, mental health care providers are regulated by health care laws in the health care sector and social work companies and the social security institute are regulated by social security laws in the social security sector [14–19].

Yet, while these different approaches traditionally are organized separately, people with mental health problems often need and receive support from both sectors [14–20]. For example, they receive cognitive therapy from mental health care providers and job skill training from providers in the social security sector. The collaboration of stakeholders from the two sectors during the application of VR interventions is therefore important. Yet, there is no uniform definition for such coordinated or integrated VR interventions. Examples of this intersectoral collaboration is nowadays more often reported and can lead to more alignment in tasks and client centeredness during vocational support [21, 22]. There is no uniform definition of collaboration within the field; collaboration can occur on different levels, and within or among different sectors [23, 24]. For example, when stakeholders from both the mental health and the social security sector work together to organize, coordinate, and develop VR interventions; cooperating on practical, financial and management aspects. Also, integration of vocational services within one specific intervention is also reported, like in supported employment (SE) or Individual Placement and Support (IPS) [25, 26]. It has been shown that interventions with integrated approaches are more successful than interventions only targeting one aspect of the vocational rehabilitation needs [13, 27, 28].

Despite these findings, collaboration between stakeholders from the two sectors during the application of vocational rehabilitation varies [23, 29]. Stakeholders experience difficulties during the implementation of VR interventions, and sub-optimal or even ineffective interventions have been reported due to insufficient collaboration (i.e., when stakeholders withdraw from the collaboration of the intervention) [29]. Insufficient VR interventions or the termination of potentially successful interventions may lead to fewer people with mental health problems gaining work or returning to work.

Although many studies on the implementation of VR interventions mention collaboration activities and the importance of collaboration between stakeholders from the mental health care and social security sectors, an overview of the actual barriers and facilitators influencing collaboration within VR interventions is missing. It is relevant to provide this overview so that we can overcome these barriers and use facilitators to improve implementation within vocational rehabilitation interventions [30].

In this study, we therefore conducted a systematic review aiming to identify barriers and facilitators influencing collaboration between stakeholders from the mental health care and social security sectors reported in studies on the implementation of coordinated or integrated VR interventions for people with mental health problems.

## Methods

### Design

We performed a systematic review of qualitative studies on the barriers and facilitators influencing collaboration between stakeholders from the mental health care and social security sectors during the implementation of coordinated or integrated VR interventions (see Table 1 for definitions). A systematic review was found to be appropriate because our aim was to provide a comprehensive, unbiased synthesis of available literature on the implementation of vocational rehabilitation [31]. Article selection consisted of four steps: 1) the primary search, 2) training the AI (Artificial Intelligence) tool to reorder the articles from step one based on relevance, 3) abstract and title screening of the reordered list, and 4) full text screening. Thereafter, we extracted barriers and facilitators influencing collaboration from the final included articles. In this review, we defined ‘collaboration’ between the social security sector and the mental health care sector as: *the situation wherein professionals or organizations from the social security sector and the mental health care sector work together within the application of a (coordinated or integrated) VR intervention.* The primary search of articles was performed on 14–04-2023.

**Table 1:** Definition of coordinated or integrated VR interventions

We defined a coordinated VR intervention as: a VR intervention where stakeholders from the mental health and social security sector collaborate on either the development of the VR intervention or organize or coordinate the VR intervention together. For example: stakeholders having agreements on practical, financial and management aspects of the VR intervention
We defined an integrated VR intervention as: a VR intervention where there is an integration of services from one sector within another sector. Such as is seen in supported employment or IPS where employment services are often integrated in mental health services

### Protocol and registration

The review was registered in PROSPERO (PROSPERO ID: CRD42023404823) [32]. We used the PRISMA guidelines for reporting systematic reviews, which is added as appendix 1 [33].

### Search and screening

#### Primary search

The primary search was performed in databases consisting of literature from various disciplines: Medline PubMed (*n* = 11,511), Web of Science (*n* = 4,821) and PSYCINFO (*n* = 368) by YN and GB and resulted in 11,783 unique articles, after duplicates were removed. Key words on *vocational rehabilitation*, *mental health care sector and social security sector* and *implementation* were used. All key words and terms used in the primary search can be found in appendix 2. Terms related to collaboration were not included in the primary search because these were not necessarily mentioned in title or abstract [34].

### Inclusion and exclusion criteria for screening

Studies published from 1985 until the final search date were included. This was assumed to be an appropriate time period, since from 1985 the introduction of VR interventions took a flight, moreover, since then the focus on implementation increased [35, 36]. The following criteria were used for inclusion: studies needed to (1) have full text articles available in English or Dutch; (2) have been published in 1985 or later; (3) have been published in a peer-reviewed journal; (4) include a qualitative evaluation like interviews or focus groups (only qualitative data or in combination with quantitative data); (5) include results reported on facilitators or barriers influencing collaboration during the implementation of coordinated or integrated VR intervention (see Table 1 for our definition of coordinated or integrated VR interventions), targeting people with mental health problems; (6) have participants directly involved in the implementation like directly involved professionals, managers or directors. Also including participants of the VR intervention; and (7) have involved stakeholders or professionals from both the mental health care sector and the social security sector during implementation.

The following publication types were excluded: (1) trial studies like randomized controlled trials (including hybrid designs), as we wanted to ensure real life practice as much as possible; (2) quantitative studies, like cost-effectiveness studies; (3) review articles; (4) conference abstracts; (5) editorials; (6) biographies; (7) letters; (8) directories; (9) commentaries, and (10) book chapters.

### Screening process

**Fig. 1:**
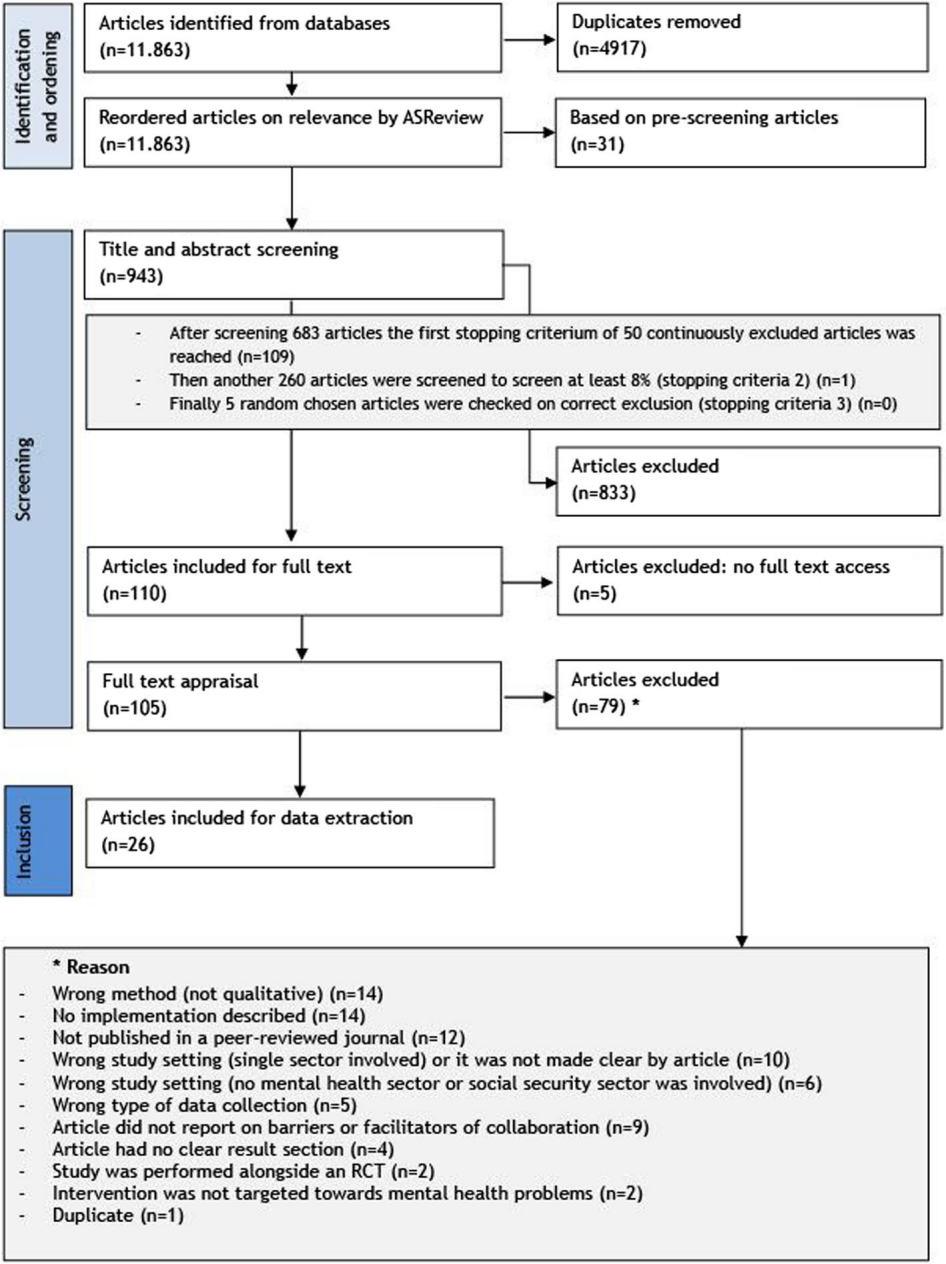
Flowchart of inclusion and exclusion of articles

Part of the screening process was assisted with AI. During every step of the screening process researcher YN and AM met regularly to discuss any doubts or disagreements. *Three stopping criteria were used to minimize the risk of missing relevant articles during the screening process*. The other researchers (FvN, JRA and MH) were consulted when no consensus was met between YN and AM. Abstracts with unclear methods sections (e.g. unclear participants or unclear data collection method) were included for full text appraisal. See also Fig. 1 for an overview of the article selection procedure.

#### Training the AI tool ASReview to reorder the articles based on relevance

As the search entailed many broad terms, a high number of articles was retrieved. Therefore, ASReview, an AI-driven screening tool, was used to support researchers during the screening process. ASReview reorders the included articles by active learning [37]. Thereby putting the most relevant articles on top and the least relevant down the list based on an algorithm created through the researchers’ input of relevant and irrelevant articles (researcher in the loop process) [34, 37–39]. Two researchers (YN and AM) independently determined article relevance by screening the abstracts and titles of 31 articles, based on the inclusion and exclusion criteria. First, six articles were singled out manually by YN and classified as “relevant” to ASReview (in consensus with AM). Then ASReview randomly selected 25 articles which were screened by YN and AM separately. Of these 25, 22 were classified as irrelevant and three as relevant. In three cases the researchers were in doubt and discussed together to reach consensus. How the model was trained (the actual classification of these 31 articles) can be found in appendix 2. After each subsequent decision whether an article was relevant or not, the algorithm was trained to reorder the article dataset, thereby optimizing the algorithm [34, 38]. After classifying 31 articles, the ASReview tool generated a new ranking predicting the relevance of all 11,783 articles in the database. This new ranking, *with the most relevant articles at the beginning of the list,* was then loaded within the RAYYAN online tool (rayyan.ai).

#### Abstract and title screening for inclusion or exclusion

The *reordered ranking* of all 11,783 articles was used to screen titles and abstracts, starting from the beginning of this list using the RAYYAN online tool. The same two researchers independently screened, based on the same inclusion and exclusion criteria. Three stopping criteria to determine when screening could end were used, minimizing the risk of missing relevant articles. After every 50–100 articles, YN and AM came together to discuss outcomes and reach consensus. In case of doubt or disagreement a third (MH) or fourth (FvN) reviewer was included in decision making. At the end of the screening process, a fifth researcher (HA) was involved in the final decision on five articles where YN and AM were in doubt. After screening 683 articles in Rayyan, the first stopping criterion was reached: 50 articles in a row were excluded [38]. After this, YN and AM continued screening until article 943 (which was 8% of the total articles in the database), the second stopping criterion was reached [38]. In the latter 260 articles, only one article was included. See also Fig. 1 on the screening process. A final extra check for correct exclusion was done on 5 random articles in the full list, moreover the third and last stopping criterion was reached.

#### Full text screening

Finally, 105 articles were included for full text screening. YN and AM again screened the full texts separately and met three times to discuss outcomes and reach consensus. Five articles were discussed with researcher MH. Seventy-eight articles were excluded after full text screening. An overview of the reasons for article exclusion can be found in Fig. 1.

In total nine articles were excluded as they did not report on barriers and facilitators for collaboration in their results section: In five articles, collaboration was not reported as an outcome in the results section [40–44]. Two articles only described collaboration as a part of implementation activities [45]. Two other articles reported collaboration as a barrier or facilitator for the implementation of the coordinated or integrated VR intervention [46, 47]. See also Fig. 1 for the flowchart of the screening process. Terms which researchers considered to be about collaboration are listed in appendix 3.

### Data extraction

In total, 26 articles were included for data extraction. Data extraction was performed by two researchers (YN and AM) based on a predesigned table. The table was set up based on agreement with involved researchers FN and MH. Two categories of data were extracted: (1) the main study characteristics and design and (2) the study content. Study characteristics and design included author, publishing year, country, study aim, type of study, data collection method, and data collection period. The data on study content included type and description of the coordinated or integrated VR intervention, description of the target group of the intervention, stakeholders mentioned in the collaboration, main study findings, and barriers and facilitators of collaboration mentioned in the results section, including tables and quotes. Data extraction of seven randomly assigned articles was done separately by both YN and AM, of which five articles were checked for consistency of the extracted data. If data was missing this was registered as ‘not described’.

### Data analysis

An open coding session for the results sections of 10 of the 26 articles took place with YN, FN, and MH. During this session, barriers and facilitators influencing collaboration were categorized, and thereafter a codebook on these barriers and facilitators was formed. We identified a barrier or facilitator for collaboration when: a) the article was aiming to study implementation factors and clearly stated in the result section that it was a barrier or facilitator for collaboration or b) the article aimed to explore factors influencing the coordinated or integrated character of the intervention, then it was assumed the complete result section reported on collaboration factors [48]. A thematic content analysis was used [49, 50]. Barriers and facilitators were categorized into six themes, and these six themes were used to analyse the remaining articles. All extracted data was again coded by YN and AM separately based on the six themes, supported by MAXQDA software Version 2022. Codes of eight articles were checked by YN and AM for consistency. In case of doubt, fragments were discussed with the third researcher (MH).

### CASP quality assessment

A quality assessment was performed on the final included articles. The Critical Appraisal Skills Programme (CASP) is a widely used tool to rate (from 0–20) individual qualitative studies based on ten items [51, 52]. The CASP of six included studies were independently assessed by two researchers (YN and AM) and checked on consistency. Then, YN assessed sixteen articles and AM four.

## Results

### Study characteristics

Details of the study characteristics of the included studies can be found in Table 2, but this section provides a brief overview:

**Table 2:** Overview of descriptives included articles

First author; publication year	Country where study was performed	Data collection method	Participants study + data collection sample size	Number of sites involved	Name & short description of the vocational rehabilitation intervention	Target group of the intervention, as described by the article	Description of the collaboration within the VR intervention	Stakeholders from Mental health sector mentioned to be involved in the coordinated or integrated VR intervention	Stakeholders from the social security sector mentioned to be involved in the coordinated or integrated VR intervention
Al-Abdulmunem M.; 2021 [53]	Unites States of America (USA)	Qualitative: interviews	Interviews with 27 key informants: IPS trainers, IPS supervisors, and IPS specialists	15 states	Individual placement and support (IPS)	Not described explicit but: people with mental illness	Not enough information to categorize the intervention as coordinated or integrated. But collaboration between sectors was explicitly described in the result section	Mental health services	Employment services, local VR
Becker DR.; 2008 [54]	USA	Not described	Not described	9 states with a minimum of 3 sites per state	Supported employment (SE)	Not described explicit but: mental health clients	Coordinated: Implementing evidence based supported employment with close collaboration between mental health and vocational rehabilitation services	Mental health	VR services
Boeltzig H.; 2008 [55]	USA	Qualitative multi-case design: interviews and document review	Interviews with 21 individuals (lead program staff)	15 sites	One-Stop Career Centers (“One-Stops”): are designed to provide a full range of assistance to job seekers including training, career counseling, referrals, job listings, and similar employment-related services. In addition, they also provide employer services	People with mental illness	Integrated: They look at the collaborative environment between workforce development (One-Stop) and the mental health systems, where they developed one team with professionals from both sectors	Mental health agencies but they also mention: mental health service providers, state/county mental health systems of care	One-stop Career Centers but they also mention: state and local Workforce Investment Boards; employment programs; the U.S. Department of Labor’s Office of Disability Employment Policy and Employment and Training Administration disability grantees, public VR system
Bond G.; 2020 [56]	USA	Qualitative: interviews (by telephone) and public documents	42 interviews with: state and regional mental health and VR leaders, agency administrators, managed care organization managers, program leaders, trainers, leaders of advocacy groups (e.g., National Alliance on Mental Illness), researchers, IPS champions (i.e., strong advocates for adopting IPS), and other stakeholders	4 states	IPS	Serious mental illness (SMI)	Coordinated: the actual approach is not described but they describe the existence of a collaborative protocol, for example on client referrals	State mental health agencies	VR agencies
Boyce M.; 2008 [57]	UK	Qualitative: interviews	Interviews with 21 representatives from 16 sites. Where possible, both a manager and an employment support worker were interviewed at each site	5 agencies	IPS	Individuals with mental health problems	Coordinated: the actual approach is not described but the mention the existence of collaboration and contact within the implementation of the intervention. Moreover, the lack of an integrated approach was mentioned	Mental health services	Employment support agencies
Dawson S.; 2021 [21]	Australia	Qualitative: interviews	11 health professionals and employment specialists, and eleven consumers	Not stated clearly but the article describes one site	IPS	Individuals who have a diagnosable mental illness receiving tertiary mental health care	Integrated: Employment specialists were integrated within a (community) mental health team. It was not described where employment specialist comes from	Community mental health Centre	Local Disability Employment Service Provider
De Greef V.; 2021 [58]	Belgium	Qualitative: group analysis	Two small groups of stakeholders (*n* = 11 and *n* = 9) including: persons suffering from mental health problems, physicians and psychiatrists, unemployed workers suffering from mental disorders, an IPS supervisor, a lawyer, etc	Not described	Existing programs that help people suffering from mental health problems to return to work	Persons suffering from mental health problems	Coordinated: The actual approach was not described. They look at existing programs that help people suffering from mental health problems to return to work. They mention the existence of coordination practices but the lack of integration	Mental health care	Different social security sectors
Giesen F.; 2007 [29]	Netherlands	Qualitative: interviews, observation, and patient monitoring	Interviews with: IPS coaches, project leaders, clients, families of clients, mental health therapists and managers of mental health and involved consultants	4 sites	IPS	SMI, young people with psychiatric disorder and people with primary psychosis onset. (target group depending on the site)	Integrated: IPS coaches were added to mental health teams. Half of the IPS coaches were previously working in mental health other half within commercial re-integration companies	A mental health agency	Two vocational rehabilitation agencies specialized in supporting people with mental issues
Hutchinson J.; 2018 [59]	England	Mixed method. Qualitative part: interviews and focus groups	Number not explicit described but included: employment specialists; team leaders; implementation managers; community mental health team staff; community mental health team managers and clinical leaders; senior managers and leaders within the host organization; service user engagement leads; external stakeholders (commissioners, local authority representatives, voluntary sector organizations); IPS clients	6 sites	IPS	People with mental health illness	Integrated: Integration of employment specialist in CMHTs (community mental health teams) Not described where the employment specialists come from	Community mental health team, National health service trusts, local national health service providers	
Isett K.R.; 2007 [60]	USA	Qualitative (Grounded case study method): interviews	30 interviews with Commissioner’s team, state mental health staff, community providers, and vocational rehabilitation services consumers and family members; local advocacy organizations	8 states	SE (next to 3 other evidence-based interventions)		Coordinated: the actual approach was not explicit described but they described that the rehabilitation services agencies were actively involved in delivery and funding of SE (by the mental health authorities)	State mental health authorities, local mental health authorities	The state's rehabilitation services agency, rehabilitation service agency (for coordinating funding)
Johanson S.; 2020 [61]	Sweden	Embedded case study including qualitative: interviews and field notes	19 interviews with employment specialists, chief medical officers of mental healthcare services, first line mental healthcare managers, mental healthcare staff including opinion leaders, strategic planners from the county council, first line managers, and handling officers from local Social Insurance Agency and Public Employment Service offices were selected	4 units	Individual Enabling and Support (IES) developed based on SE for people with SMI Time use, motivational support, and cognitive support strategies were added to better fit the support needs of persons with affective disorders	People with affective disorders and/or long-term pain	Coordinated: Two IES employment.. specialists were recruited for the project. Each employment specialist covered two mental healthcare units but were not integrated. Each IES ES served specific teams	Mental health care	Social Insurance Agency, Public Employment Service
Johnson‑Kwochka A.; 2017 [62]	USA	Qualitative: interviews	Interviews with 52 representatives from 48 state behavioural health agencies and 55 representatives from 51 state vocational rehabilitation agencies	50 states	IPS	Not explicit described but: people receiving mental health services	Coordinated: State and regional leaders in the IPS Learning Community have collaborated on disseminating, implementing, and sustaining IPS. The learning community has a decentralized structure with two tiers: the national leadership team at the IPS Employment Center located in Lebanon, NH, and leaders from state and regional agencies who have planned, implemented, and monitored IPS programs within their states	State and regional behavioral health agencies	Vocational rehabilitation agencies
Marshall T.; 2008 [63]	USA	Qualitative: observational (Field diary entries of site visit), interviews, and fidelity assessment reports	5 fidelity assessment, accompanied by interviews with the program leaders (supervisors of the employment specialists) and observations	9 sites	SE	Clients from mental health agencies	Integrated: They assigned employment specialists to mental health agency treatment teams to enhance integration (of the clinical coordination of services). Not clear where the employment specialist come from although they were hired	8 mental health agency and one comprehensive psychosocial service unit (State) mental health authority	Vocational rehabilitation agencies, state vocational agency
Menear M.; 2011 [64]	Canada	Qualitative: document study and interviews	53 participants were interviewed: 21 employment specialists, 21 team coordinators or program managers, 2 researchers, 6 regional or provincial decision makers, and 3 actors operating at regional or provincial levels concerned by related issues	15 sites providing 20 SE services	SE	Not explicit described but: people with mental health issues	Integrated: The actual approach depends on the site but what was mentioned: supported employment service is integrated in mental health services	Depends on the site but what was mentioned: mental health teaching hospital, general hospital or regional health agencies	Depends on the site but what was mentioned: SE agencies, employment authorities and community based non-profit organizations
Moe C.; 2021 [65]	Norway	Qualitative: focus groups and field notes	45 IPS employment specialists, among them 11 IPS supervisors (both employment specialists and supervisors)	14 sites	IPS	People with mental illness	Integrated: Employment specialist from Norwegian Labour and Welfare Administration integrated in different mental health teams from the mental health agencies	The Norwegian mental health services	The Norwegian Labour and Welfare Administration
Noel V.A.; 2017 [66]	USA	Qualitative: interviews	IPS program leaders	129 sites in 13 states	IPS	Not explicit described but: clients from community mental health centers	Not enough information to categorize the intervention as coordinated or integrated. But collaboration between sectors was explicitly described in the result section	Community mental health centers Support services; including clinical and state mental health authority)	Support services including vocational rehabilitation services
Oulvey (2013) [67]	USA	Qualitative: focus groups	21 focus groups: Five of the focus groups were comprised of consumers of IPS services, five of staff serving on IPS teams, five of mental health practitioners working with IPS teams, and six of state VR counselors and VR office supervisors. All but one of the VR groups was comprised of staff working with local IPS programs. Participants were chosen on the basis of their affiliation with IPS teams from all major regions of Illinois. IPS teams, VR staff, and the persons they served from urban, suburban, and rural areas were represented in the focus groups	Not described. But this was described: rural and urban locations	IPS and VR, differences was not explicit described	Not explicit described but they mention: people with psychiatric disabilities receiving VR services IPS consumers	Integrated: the approach was not explicitly described but the consumers’ perception about the approach was that professionals worked in one team	IPS teams, mental health practitioners working with IPS teams	State VR counselors and VR office supervisors
Pogoda T.K.; 2011 [68]	USA	Qualitative: interviews	84 respondents, including supported employment staff, mental health employees and Veteran affairs medical center leadership	6 sites	SE	Not directly described but: veterans with mental health issues	Integrated: employment specialists were integrated in a Veteran affairs medical center	Veteran affairs medical center	Not described They mention employment specialists but it’s not clear from which stakeholder they come from
Rapp C.A.; 2010 [69]	USA	Qualitative: implementation monitors, interviews and field notes	Implementation monitors information during site visits and meetings ed Interviews with consumers, direct service workers, supervisors, and administrators Field notes	5 sites	SE EBP (Supported employment evidence-based principles), based on 6 principles: (1) eligibility is based on consumer choice; (2) employment services are integrated with treatment; (3) competitive employment is the goal; (4) rapid job search; (5) follow-along supports are continuous; (6) consumer preferences direct the work. Integrated Dual Diagnosis Treatment simultaneously treats the mental health and substance abuse disorders using a stage-wise approach with motivational interviewing, substance abuse counseling, self-help and other support services	Mental health and substance abuse disorders	Integrated: The teams consisted of a program leader (in some case, two supervisors shared the role) and 3–6 direct service staff Each site created a leadership Team comprised of the community mental health center executive director, Community support services director, program leader, consumers, families, and a state representative. Meetings were facilitated by the consultant and trainer assigned to the site	Community mental health centers	
Seebohm P.; 2003 [70]	UK	Qualitative: interviews	CMHT (community mental health teams): care coordinators, one community psychiatric nurse, one Occupational therapist and one social worker In the two assertive outreach teams, interviews with 1 community psychiatric nurse, and 1 social worker. One assertive outreach team did not have an occupational therapist, and the other was not available	3 CMHT sites	SE	Not explicitly described but client from mental health teams	Integrated: Depends on the site but 2 sites the CMHT had a vocational specialist (employed by the national health services trust) to work with The third site had an occupational therapist, supported by a vocational specialist (employed by the social services) within each community mental health teams Not made clear where the vocational specialist and occupational therapist came from	Community mental health teams sites	The local National Health Services trust, social services
Sharek D.L.; 2020 [71]	Ireland	Qualitative: focus groups	3 qualitative, semi-structured focus groups with IPS Employment Specialists (ES) and Occupational Therapist Managers	Not described	IPS	Clients of 'mental health services'	Integrated: As part of the program, IPS Employment Specialists were recruited from employment support organizations outside of the mental health services and their work was supervised by occupational therapist managers within mental health services, so that IPS could be integrated across the employment and health fields	Multi-Disciplinary Team in the Irish mental health services	Specialists were recruited from employment support organizations
Swanson S.J.; 2014 [72]	USA	Qualitative: Interviews, on-site observations and discussion	Not described	3 sites (= states)	IPS	Mental health care clients under treatment	Integrated: the actual approach differs per state but: creating teams of mental health and employment practitioners by assigning an employment specialist or VR counselor to a mental health center Also they mentioned cross-agency steering groups	Mental health agencies	Differs per state but: employment agencies or community rehabilitation providers
Van Erp N.H.J.; 2017 [73]	The Netherlands	Qualitative: interviews, observations and examination of records	Interviews with program leaders, clinical leaders, managers, and the trainer	4 sites	SE	People with severe mental illness (SMI)	Integrated: Employment specialists from vocational services were assigned to mental health teams	Mental health organization	Vocational services
Vukadin M.; 2018 [74]	The Netherlands	Qualitative: interviews	15 interviews with representatives from one municipality, two different UWV front offices, two different locations of one Mental Health Agency And one health insurance company	2 sites	IPS	People with severe mental illness (SMI)	Coordinated: Regular meetings between the different stakeholders involved Regular meetings between IPS employment specialists (from mental health agencies) and vocational rehabilitation professionals from the involved agencies	Two mental health agencies and a health insurance company	The Dutch Social Security Institute (SSI), the municipality of Amsterdam
Vukadin M.; 2021 [75]	The Netherlands	Qualitative: interviews and focus groups	10 interviews with IPS clients and 2 focus groups with IPS employment specialists	2 sites	IPS	Mental health care clients under treatment (SMI, see also Vukadin 2018)	Coordinated: Regular meetings between the different stakeholders involved Regular meetings between IPS employment specialists (from mental health agencies) and vocational rehabilitation professionals from the involved agencies	Two mental health agencies and a health insurance company	The Dutch SSI, the municipality of Amsterdam
Wihlman U.; 2008 [76]	Sweden	Qualitative: interviews and focus groups	51 individual interviews and 68 persons were involved in 14 focus groups. With involved professionals and directors from: social insurance offices; health care; social services and employment services	Number of sites unclear but: 16 different inter-professional teams	Not clear: at least two organizations involved in the VR of individuals within the target group	People with multi or complex problem including unemployed people with psychiatric disorders	Coordinated: At least two organizations were involved in the implementation of the VR intervention with a formalized relationship aimed at practical and financial coordination.	The local health services	The social insurance office, the municipal social service, the office of the state employment service

We included 26 articles. Of these, 21 articles reported on the implementation of SE and, specifically, 14 articles were on IPS. An additional 5 studies were about another vocational rehabilitation intervention, of which 2 mentioned being related to the principles of SE or IPS. One study did not look at one intervention in specific but was on the current practice of VR in Belgium [77]. One study had no clear description of the vocational rehabilitation intervention of interest [76]. Two interventions were clearly described as the implementation of newly created collaborative teams, eleven studies mentioned collaboration agreements involving agencies from both sectors while eight studies mentioned that the collaboration was made via the integration of a supported employment specialist into mental health teams. Finally, five studies did not describe the collaborative set-up of the intervention [53, 56, 57, 67, 77].

Most of the studies were conducted in the United States of America (USA) (*n* = 11) or Europe (*n* = 13), though one study was from Canada and one from Australia. Participants included in the study were mostly professionals from multiple levels (like SE or IPS specialists, mental health professionals, managers, program leaders or directors). Sixteen studies specified that professionals from both sectors were participating. In the other ten studies this was not the case or not made clear, though these studies still presented results on collaboration between sectors. Moreover, seven studies involved participants (clients) from the VR intervention [29, 59, 60, 67, 69, 75, 77] and two studies did not describe the participants [54, 72].

Finally, four included studies were only examining and reporting on *barriers* for collaboration during the implementation of the coordinated or integrated VR intervention [63, 68, 69, 76], which might incorporate bias.

### CASP outcomes

All included studies were rated (from 0–20) using the CASP checklist regarding the reporting quality of the evidence. We added this filled checklist as appendix 4. We found one study with a reporting quality of 8, six with an reporting quality between 10–15 and nineteen with a reporting quality above 15. Overall seen, articles scored relatively low on reporting on *recruitment strategy*, *relationship between researcher and participants* and *ethical considerations*.

### Describing results on collaboration barriers and facilitators

#### Themes of barriers and facilitators

We thematically organized the barriers and facilitators to collaboration found in the literature and identified six themes: attitude and beliefs, engagement and trust, governance and structure, practical issues, professionals involved, and including client-centeredness. Table 3 provides a short description of every theme and gives an overview on which theme was found in which included article, and whether this was a barrier or facilitator.

**Table 3:** Overview of barriers, facilitators that influence collaboration during the implementation of coordinated or integrated VR interventions

Theme	Subtheme	Reported as barrier by:	Reported as facilitator by:
**Theme 1: Attitudes and beliefs** *Feelings and opinions about the collaboration from the individuals who are directly or indirectly involved*	Importance of attitudes and beliefs	[57, 58, 68, 69, 74, 76]	[61, 73]
	Collaboration activities related to attitudes and beliefs	[58, 65, 68, 73, 76]	[54, 59, 65, 70, 71]
	Attitude and beliefs of others involved	[54, 56]	[74]
**Theme 2: Engagement and trust** *Engagement: The amount of feeling from individuals about being actively part of the collaboration* *Trust: A relationship between professionals where they believe in the other and have mutual understanding and respect*	Importance of engagement:	[29, 57, 61, 71]	[61, 65, 66]
	Feeling of trust and ownership for engagement	[61, 65, 71, 76]	[70, 71, 74]
	Collaboration activities influencing engagement	[76]	[61, 72, 74]
**Theme 3: Governance & structure** *The way that the collaboration and involved organizations are structured and managed*	Agreements structure/importance of clear structure	[56, 61, 68, 69, 71, 74–76]	[56, 58, 62, 70–72]
	Dealing with multiple stakeholders	[60, 61, 65, 75, 76]	[58, 62]
	New and existing structures	[61, 65]	[56, 65, 72, 76]
	Funding & financial incentives	[58, 76]	[61, 72, 76]
**Theme 4: Practical issues** *Practical issues regarding the implementation of the collaboration*	Practical issues regarding systems and time	[59, 65, 68]	[21, 61, 63, 71, 72]
	Co-location	[29, 53, 55, 59, 65, 68]	[21, 53, 59, 72, 74, 75]
	Legislation	[61, 65]	[58, 65]
**Theme 5: Professionals involved** *Professionals that directly or indirectly participate in the implementation of the collaboration*	Key persons	[76]	[55, 71, 72, 74, 75]
	Characteristics and personality	Not reported	[61, 64, 66, 71]
	Continuity of involved individuals	[61, 74–76]	[64, 67]
**Theme 6: client-centeredness** *When helping the client is the main goal for the collaboration*		[61, 76]	[21, 70, 75]

#### Theme 1: Attitudes and beliefs

Within this first overarching theme on the attitudes and beliefs towards the collaboration or integrated approaches, three subthemes were found: 1) the importance of attitudes and beliefs; 2) behaviour related to attitudes and beliefs, and 3) attitudes and beliefs of others involved.

##### Importance of attitudes and beliefs

Multiple articles presented the importance of attitudes and beliefs from professionals involved in the collaboration; these could either be a barrier or facilitator for the collaboration [61, 68, 73, 74, 76]. Articles described that this was most often mentioned by professionals on the operational level of the collaboration [68, 73, 74]. But some articles also reported this on the level of involved organizations; when values and beliefs conflicted between collaborating stakeholders, this hindered collaboration [57]. It was not always clear exactly what kind of attitudes of beliefs were being referred to [57]. But some articles mentioned specifically that differing beliefs on what was best for clients during: mental health professionals did not always believe in the value of employment or vocational recovery integrated within treatment, leading them to refuse to collaborate. For example by refusing to communicate about clients [58, 68, 69, 74, 77]. Other articles mentioned that when people involved in the collaboration had positive attitudes towards the intervention and the use of work within mental health rehabilitation it facilitated better collaboration [59, 61, 73].

##### Collaboration activities related to attitudes and beliefs

Multiple articles reported that lack of knowledge about collaboration or the idea behind the integrated intervention model made professional reluctant to collaborate [58, 68, 73]. One article explained this phenomenon: a lack of understanding of mental health professionals led to not seeing the importance of the collaboration, leading to refusal to collaborate [65]. Meanwhile, other articles revealed that recognizing own’s lack of knowledge, sharing knowledge and success stories with each other stimulated collaboration, helping folks to overcome resistance and making attitudes toward the collaboration more positive [54, 65, 70]. Finally, a feeling of territoriality between sectors was mentioned by articles as hindering collaboration, because organizations and individual professionals from the social security sector were guarding their own target groups or caseloads [76].

##### Attitude and beliefs of others involved

Moreover, articles demonstrated that professionals involved in the coordinated or integrated VR interventions did not always feel supported by colleagues from their own organizations, due to these colleagues holding negative attitudes of these colleagues [74]. Thus, the lack of belief in and knowledge about the collaboration of other colleagues can also work as a barrier for collaboration. Additionally, lack of a governmental statement on the importance of integration of services was mentioned as a barrier in one of the studies [54, 56].

#### Theme 2: Engagement and trust

##### Importance of engagement

The reviewed articles presented engagement from professionals of all organizations involved in the collaboration as an important factor for success [29, 57, 65, 66]. Articles showed this on all levels — not only engagement and trust from executive professionals in the integrated intervention or collaborative teams, but also at the level of managers and opinion leaders [61, 66, 71]. How this engagement can be achieved was not always made clear [57].

##### Feeling of trust and ownership for engagement

In some articles mutual trust and a feeling ownership within the collaboration was shown to be important to achieve collaboration engagement [58, 61, 74, 76]. Again, articles mentioned this to be important at all levels [61, 76]. Moreover, building trust was mentioned to be necessary for collaboration, which takes time [71]. One article explained that mutual trust increased open communication and reliance on each other’s opinion and expertise [70, 74]. Regarding the feeling of ownership, articles reported that having mandate and influence and ‘feeling seen and heard’ by the other party contributed to successful collaboration [74]. On the other hand, if the other party does not show ownership, the SE professional feel lonely and alienated, which was mentioned as a barrier for collaboration [65, 76].

##### Collaboration activities influencing engagement

Studies revealed that stakeholders’ commitment and thus collaboration was increased by the engagement of decision makers and an active contribution of all involved parties during discussions [72, 74]. Articles reported that engagement can be stimulated by providing the following: having an independent process leader, an active contribution of organizing activities, and having decision makers with the right mandates involved at the decision-making level [61, 74]. Some studies suggested that all of these factors increase the feeling of ownership and thereby the engagement in the collaboration [74, 76]. Subsequently, having different goals and perspectives on the collaboration by the decision makers was hindering engagement, and thus collaboration [76].

#### Theme 3: Governance & structure

##### Importance of clear agreement structure

On all levels, studies reported that a lack of clarity about tasks and responsibilities, ‘who does what and when,’ hindered collaboration [61, 68, 69, 74]. It was mentioned that uncertainty about ‘who is leading who’ led to power struggles between management and advisory board [76]. In contrast, a clear management structure for collaboration was reported to increase trust on an operational level, reassuring the quality of governance [71]. Examples that were presented as facilitating collaboration were: a manager who is visible in the team and who ensures a clear distribution of roles, the right competencies of the employment specialist on the team, clear governance, and finding solution for problems, such as privacy issues [71]. At the same time, unclear agreements were mentioned to lead to miscommunication and insecurities (and again: distrust) among the people involved [76]. The studies also revealed that collaboration was hindered when it was unclear which clients the professionals in the collaboration were working for [76]. Clear governance involving both sectors with clear roles and tasks provided in, for example, a collaboration protocol with working arrangements was mentioned to be a facilitator for collaboration [56, 62, 70, 71].

##### Dealing with multiple actors

Organizing a collaboration, including financial coordination, was mentioned to be difficult when many people and stakeholders were involved [60, 61, 75, 76]. Building a network structure was mentioned to facilitate collaboration [58, 62]. On the other hand, articles showed that professionals involved in collaborations found it difficult to balance time between all those actors, in order to please everyone [61, 65].

##### New and existing structures

Multiple articles showed that an already existing positive history of collaboration was mentioned to be a facilitator for collaboration [56, 65, 72]. In line with this, building a stakeholder network was presented to be facilitating factor [61]. Without any history of collaboration, one of the articles mentioned that it helped to start the collaboration with a newly created multidisciplinary team, while merging or integrating services in already established teams was presented as barrier [76]. This was illustrated by another article which mentioned that new individuals in a collaboration often get stuck in existing structures because they are not familiar with these [65].

##### Funding & financial incentives

Funding of the collaboration or integrated intervention was mentioned by articles to be influence the collaborations’ success [61, 72, 76]. Where an established clear procedure was found to be stimulating collaboration, even as joint funding [61, 72]. But on the other hand, it was reported by articles that not wanting to share resources, and—on the other hand—not getting paid for participation within the collaboration were actually hindering collaboration [77]. Funding was presented as being related to engagement by one article in two ways: On the one hand, it is a barrier for engagement and collaboration if one of the parties doesn’t feel a financial incentive. On the other hand, not contributing financially (in this case by the social security sector) makes that the other party does not see you as an owner of the intervention, which was presented as hindering the collaboration [76].

#### Theme 4: Practical issues

##### Practical issues regarding systems and time

Practical barriers for collaboration that were mentioned in the articles were: bureaucracy, working in two different systems, difficulty with matching the agendas of two organizations (resulting in not being able to be present in all team meetings), and not having access to each other’s systems, which made professionals dependent on each other to receive access [29, 61, 65, 68, 72]. Insufficient time to get used to each other was also mentioned as a barrier [68, 71]. Some articles reported that these barriers were appointed to the own organization, such as not being given time to collaborate and prioritizing internal organization meetings and high caseloads, but this was not always the case [71, 72]. Other articles showed that project leaders or managers could facilitate collaboration by making structural changes around these practical issues, such as by providing access to systems, reducing double paperwork, reducing production standards, or appointing dedicated professionals to the collaboration [63, 72].

##### Co-location

Both working in the same office and having weekly meeting were mentioned by multiple articles as increasing collaboration success by providing more alignment in case management, informal discussions and sharing knowledge, and accessibility of involved professionals [21, 55, 59]. Colocation was not only cited as solving practical collaboration issues but was also shown to contribute to building trust between professionals from different organizations [74]. In line with this, having limited time for meetings and informal discussions (reported by both mental health and social security professionals), physical distance between organizations leading to a literal separation, not being visible and having less contact were presented as barriers [29, 53, 59, 68].

##### Legislation

Legislation was reported in multiple articles as a practical barrier to collaboration, prohibiting professionals from sharing information, accessing systems or even having team meetings [58, 61, 65]. But it was also made clear by these articles that this issue was resolved by involved managers who worked to find solutions: making rules or user agreements on this issue, providing system access, making a 0% working contract for colleagues from the other organization to be allowed to enter meetings officially [65]. Some articles described that professionals did not share information with professionals from the other sector due to confidentiality reasons, mostly argued by mental health care professionals, but other articles showed that this might be more a trust issue than a practical issue [61, 77]. Again, managers who addressed and reassured employees on these concerns facilitated better collaboration [65, 71].

#### Theme 5: Professionals involved

##### Key persons

Key point people within each involved organization were described to be facilitators for collaboration [55, 75]. Some articles stated that these key people should be easy to reach, answering questions and providing short (contact) lines [55, 74]. It was also reported that an independent project leader could facilitate collaboration, and that the project leader should not be employed by one of the involved stakeholders as this could result in trust issues [72]. One article emphasized that the project leader should also not be too close with politics, as this can also lead to trust issues [76].

##### Characteristics and personality

Characteristics, personality, and skills of supported employment specialists were mentioned to be facilitators [64, 66, 71]. Although one study did not reveal what these characteristics, personality, and skills should be specifically, though the article reported that a positive mindset from new individuals in a team was helpful for the collaboration [71]. An enthusiastic and supportive management was also to mentioned to be a facilitator for collaboration [61, 66].

##### Continuity of involved individuals

Another issue related to involved individuals that arose from the articles was the existence or lack of continuity of staff, seen in both sectors [64, 67, 75]. For example, due to high staff turnover within the integrated intervention but also internally within organizations [61, 75]. Changes in management were also mentioned as influencing the collaborations negatively when new managers curtailed involvement in the collaboration [61]. Likewise, an unclear vision of the collaboration was mentioned to lead to constantly forming new groups making the collaboration unstable and bureaucratic [76].

#### Theme 6: client-centeredness

The final overarching theme that was found was client-centeredness. Having a multidisciplinary, client-centred approach, for example via co-location, was reported in the articles to facilitate the actual collaboration [21, 67, 70]. Moreover, this client-centeredness being missing was seen as a barrier [61, 76]. Other articles suggested that having clear collaboration rules and explaining these rules to the client was also helpful in collaboration between professionals [75]. Finally, it was presented that focus on formal and financial aspects was a barrier for the collaboration, since it distracts from client perspectives [76]. While client-centeredness stimulates participation of professionals involved [21, 76].

## Discussion

In our systematic review we aimed to identify barriers and facilitators for collaboration between stakeholders from the mental health and social security sectors in the implementation of coordinated or integrated VR Interventions. Barriers and facilitators were clustered around six main themes: attitudes and beliefs, engagement and trust, governance and structure, practical issues, professionals involved and client-centeredness.

### Trust issues

We found that having a positive attitude and trust toward the collaboration were important facilitators for collaboration, whereas a negative attitude and a lack of trust were mentioned as barriers. A similar finding was presented in a study on the implementation of VR interventions in the social security sector [78]. Likewise, Nauta et al. [79] found that trust and attitude influenced collaboration between professionals working on vocational recovery in another (comparable) care sector, namely occupational health services. Thus, it seems that trust issues play a crucial role in collaboration between sectors, not necessarily the specific sectors that were studied in the present review. In the present review, a negative attitude and lack of trust were more often mentioned for mental health care professionals than for employment professionals. In the included articles in our review, this was explained by mental health care professionals being hesitant to integrate work into treatment for several (stigmatic) reasons: they believed work might not help in recovery or that people with mental health problems are unable or uninterested in working [58, 74, 76]. Moreover, the papers reported that professionals think that the views of the social security sector might be conflicting with the mental health care sector’s goals [58, 76]. This is in line with other research on mental health professionals’ perspectives regarding the employability of people with mental health problems [7, 80–82]. The lack of knowledge on the benefits of work for people with mental health problems possibly contributes to this; mental health professionals might perceive a higher trust in their own knowledge than other ones’ knowledge. This is again in line with the findings of Nauta et al., who also found that knowledge-based trust issues between general practitioners (GP’s) and occupational health professionals (OP’s) within occupational health services hindered their collaboration [79, 83]. Nauta et al. [83] found that trust can be built via equalizing status increasing common knowledge (via an integral course) and stimulating professional contacts, which is in line with our review findings. Our review showed sharing successes can also contribute to improved collaboration.

### Organisational aspects and perceived barriers

In the present review, we found that difficulties in trust and engagement among involved professionals also seemed to be related to the organizational structure of the coordinated or integrated VR intervention. Integrating an individual from one sector (often from the social security sector) into already existing structures from another sector (the mental health care sector) may hinder collaboration because it seems to contribute to a lack of trust and engagement of the professionals involved [65]. The organizational structure of VR interventions is related to the context of the country in which the VR intervention is implemented. National or even regional differences like law-structures likely impact the difficulty of integrating services. Moreover, these differences might influence professional perspectives on the collaboration. This advocates for a deeper understanding of how ‘the bigger systems’ influences the collaboration and facilitators and barriers at different levels. An analysis of contextual factors influencing the perspectives of collaboration would add to the knowledge of this scientific field. Yet, the minimal information that articles provided on the exact context complicated this. However, we have no indication that the collaboration themes and the facilitators and barriers mentioned differed between countries. This aligns with an article arguing that we need more insight in the organizational aspects and the perception of perceived barriers regarding implementing performance of programs such as VR interventions [30]. Moreover, it has previously been described that organizational (or more practical) barriers of collaboration can also be explained by the subjective perception of the involved professionals rather than the objective description of the barrier itself [84]. Following this view, it would be useful to design implementation strategies based on behavioural change theories to influence these subjective perceptions on collaboration [84]. A potential implementation strategy to do so could be finding a mutual goal. This is also supported by our previous study, in which we found that improving client perspectives regarding mental health and participating in work was an important driver for professionals to collaborate [59]. During this process of finding a shared goal process managers should align on the beliefs and ideas of the professionals involved.

### Strengths & limitation

Whereas many studies have mentioned that collaboration in itself is an important facilitator or barrier for the implementation of VR interventions [10, 46, 62], the strength of this review is that we focused on the actual barriers and facilitators for collaboration between stakeholders from two sectors, during the implementation of coordinated or integrated VR interventions. The use of an AI induced tool within this review can also be seen as a strength, since it allowed us to use a broad seeking scope during the primary search, which generated many potential relevant articles, yet made it feasible to go through them in a reasonable amount of time [38, 85]. At the same time the use of an AI tool has some limitations, like the risk of missing relevant articles because not all articles were screened by the researchers themselves. We built in several steps to adjust for this potential bias, including multiple stopping criteria making the search more hybrid and robust [34, 38, 85, 86].

Reviewing only qualitative studies may be considered a limitation. We decided to only focus on studies with a qualitative character, as they provide a deep understanding of factors as perceived by the interviewees. Nevertheless, we may have missed aspects of collaboration that could have been mentioned in quantitative studies. However, the many qualitative studies that were included in the present review gave such a broad overview of themes of facilitators and barriers, that we do not suspect that major themes were missed. Reviewing qualitative studies has some challenges, like being dependent on the quality of included articles and having a distance between the reviewers and the original participants of the included studies, which might lead to interpretation difficulties [87]. Still, we managed to optimize quality by having all analytic steps completed by two researchers separately, having multiple discussions among all involved researchers, and checking included articles for quality with the CAPS checklist [87].

### Practical implications

This review provides many practical barriers and facilitators that can be used to improve collaboration in existing interventions and in the development of new VR interventions. Building trust seems to be key, and based on the facilitators that came forth from our review, possible ways to acquire this are: co-locate involved individuals to learn from each other and build trust, formalizing the collaboration with clear agreements, having the collaboration led by an independent project leader, and increasing knowledge about each other’s expertise and field. Moreover, we revealed that practical barriers and facilitators were not always actually present but were sometimes also based on the perception of professionals. Therefore, it is important in setting up a collaboration to get more insight into the perception of professionals about collaboration. Concrete takeaways include using the benefits of sharing knowledge on the use of work for people with mental health problems, finding a shared goal, sharing successes between professionals from both sectors. This can be achieved via a multidisciplinary, client-centered approach, for example via a practical establishment of co-location of professionals. We would suggest that providing a complete overview of strategies would be an interesting next step following up this research.

### Implications for further research

During the review process we found and excluded articles that mentioned the importance of collaboration without analysing the actual influential factors, i.e. facilitators and barriers, for collaboration. Moreover, details were not always given in the articles regarding, for example, what kind of attitudes or trust issues hindered collaboration. Articles likewise often used poorly defined concepts and umbrella terms, like engagement, commitment and collaboration, which could be easily understood a number of ways. We therefore suggest future studies and articles to report more explicitly the meaning of these concepts.

Moreover, articles were often not clear about the causality of factors. Although this review revealed insights in collaboration and factors that impact collaboration in the specified context, it is recommended to report on factors influencing collaboration more specifically. For example, the field would benefit from more insight into the interaction of the perception of professionals regarding the barriers and facilitators towards collaboration rather than questioning practical barriers and facilitators. The role of management as an intermediate factor in processing these collaboration aspects among professionals could contribute to the field of knowledge on complex interventions, such as coordinated or integrated VR interventions.

## Conclusion

Six main themes of barriers and facilitators for collaboration between stakeholders from the mental health care and social security sectors were found in this review: attitudes and beliefs, engagement and trust, governance and structure, practical issues, professionals involved, and client-centeredness. We found that positive attitudes and beliefs towards the coordinated or the integrated approach can enhance collaboration, whereas a negative attitude and lack of trust can hinder it. Often practical barriers were mentioned for collaboration, but these did not always exist and could be solely based on perceptions of professionals. Collaboration between stakeholders from different sectors could be increased by improving positive attitudes and mutual trust and increasing knowledge about each other’s expertise. Along with highlighting the benefits of work for people with mental health problems, sharing success stories, co-location of professionals, and having a clear governance.

## Supplementary Information

Supplementary Material 1: PRISMA Guideline for reporting systematic reviews.

Supplementary Material 2: Full search strategy including AI training model.

Supplementary Material 3: Terms considered to be on collaboration.

Supplementary Material 4: Complete CASP checklist score.

## Data Availability

The datasets generated and/or analysed during the current study are publicly available and are available as appendixes of this article or available on request.

## Abbreviations

AI: Artificial intelligence
Casp: Critical Appraisal Skills Programme
CMHT: Community mental health team
IES: Individual Enabling and Support
IPS: Individual Placement and Support
PRISMA: Preferred Reporting Items for Systematic reviews and Meta-Analyses
SE: Supported employment
SE EBP: Supported employment evidence-based principles
SMI: Severe mental illness
USA: United states of America
UWV: The Dutch Social Security Institute: The Institute for Employee Benefits Schemes
VR: Vocational Rehabilitation
