# Supplementary material for: Barriers and facilitators of collaboration during the implementation of vocational rehabilitation interventions: a systematic review
Source: BMC Psychiatry. 2024 Nov 1;24:759. doi: 10.1186/s12888-024-06223-y (PMC11529217; doi:10.1186/s12888-024-06223-y)
Supplement: Supplementary file 2 — Supplementary Material 2: Full search strategy including AI training model. [file 12888_2024_6223_MOESM2_ESM.docx]

# Step 1: Final PRIMARY Search 13/04/2023

Title: Barriers and facilitators of collaboration during the implementation of vocational rehabilitation interventions: A systematic review.

## PubMed (11,511)

| **Search** | **Query** | **Results** |
| --- | --- | --- |
| #4 | Search: **#1 AND #2 AND #3** | [11,511](https://pubmed.ncbi.nlm.nih.gov/?term=%231+AND+%232+AND+%233&sort=relevance&ac=no) |
| #3 | Search: **"Rehabilitation, Vocational"[Mesh] OR "Employment, Supported"[Mesh] OR Vocational*[tiab] OR Employment*[tiab] OR "Individual placement and support"[tiab] OR "return to work"[tiab] OR occupation*[tiab]** | [279,978](https://pubmed.ncbi.nlm.nih.gov/?term=%22Rehabilitation%2C+Vocational%22%5BMesh%5D+OR%0A%22Employment%2C+Supported%22%5BMesh%5D+OR%0AVocational%2A%5Btiab%5D+OR%0AEmployment%2A%5Btiab%5D+OR%0A%E2%80%9CIndividual+placement+and+support%E2%80%9D%5Btiab%5D+OR%0A%E2%80%9Creturn+to+work%E2%80%9D%5Btiab%5D+OR%0Aoccupation%2A%5Btiab%5D&sort=relevance&ac=no) |
| #2 | Search: **"Social Security"[Mesh] OR "Health Services"[Mesh] OR "health agenc*"[tiab] OR "health service*"[tiab] OR "health staff"[tiab] OR "service staff"[tiab] OR "employment service*"[tiab] OR "insurance service*"[tiab] OR "welfare service*"[tiab] OR "vocational service*"[tiab] OR "Social Insurance*"[tiab] OR "social securit*"[tiab] OR "rehabilitation service*"[tiab] OR "community service*"[tiab] OR Employment-service*[tiab] OR "community mental health*"[tiab]** | [2,524,795](https://pubmed.ncbi.nlm.nih.gov/?term=%22Social+Security%22%5BMesh%5D+OR%0A%22Health+Services%22%5BMesh%5D+OR%0A%E2%80%9Chealth+agenc%2A%E2%80%9D%5Btiab%5D+OR%0A%E2%80%9Chealth+service%2A%E2%80%9D%5Btiab%5D+OR%0A%E2%80%9Chealth+staff%E2%80%9D%5Btiab%5D+OR%0A%E2%80%9Cservice+staff%E2%80%9D%5Btiab%5D+OR%0A%E2%80%9Cemployment+service%2A%E2%80%9D%5Btiab%5D+OR%0A%E2%80%9Cinsurance+service%2A%E2%80%9D%5Btiab%5D+OR%0A%E2%80%9Cwelfare+service%2A%E2%80%9D%5Btiab%5D+OR%0A%E2%80%9Cvocational+service%2A%E2%80%9D%5Btiab%5D+OR%0A%E2%80%9CSocial+Insurance%2A%E2%80%9D%5Btiab%5D+OR%0A%E2%80%9Csocial+securit%2A%E2%80%9D%5Btiab%5D+OR%0A%E2%80%9Crehabilitation+service%2A%E2%80%9D%5Btiab%5D+OR%0A%E2%80%9Ccommunity+service%2A%E2%80%9D%5Btiab%5D+OR%0AEmployment-service%2A%5Btiab%5D+OR%0A%E2%80%9Ccommunity+mental+health%2A%E2%80%9D%5Btiab%5D&sort=relevance&ac=no) |
| #1 | Search: **"Implementation Science"[Mesh] OR "Health Plan Implementation"[Mesh] OR "Delivery of Health Care, Integrated"[Mesh] OR Implement*[tiab] OR Integration*[tiab] OR deliver*[tiab] OR sustain*[tiab] OR Barrier*[tiab] OR Facilitator*[tiab]** | [2,351,164](https://pubmed.ncbi.nlm.nih.gov/?term=%22Implementation+Science%22%5BMesh%5D+OR%0A%22Health+Plan+Implementation%22%5BMesh%5D+OR%0A%22Delivery+of+Health+Care%2C+Integrated%22%5BMesh%5D+OR%0AImplement%2A%5Btiab%5D+OR+%0AIntegration%2A%5Btiab%5D+OR%0Adeliver%2A%5Btiab%5D+OR%0Asustain%2A%5Btiab%5D+OR%0ABarrier%2A%5Btiab%5D+OR%0AFacilitator%2A%5Btiab%5D&sort=relevance&ac=no) |

## Web of Science (4,821)

| **Search** | **Query** | **Results** |
| --- | --- | --- |
| #4 | **#1 AND #2 AND #3** | 4,821 |
| #3 | **TS=(Vocational* OR Employment* OR "Individual placement and support" OR "return to work" OR occupation*)** | 491,268 |
| #2 | **TS=("health agenc*" OR "health service*" OR "health staff" OR "service staff" OR "employment service*" OR "insurance service*" OR "welfare service*" OR "vocational service*" OR "Social Insurance*" OR "social securit*" OR "rehabilitation service*" OR "community service*" OR Employment-service* OR "community mental health*")** | 206,949 |
| #1 | **TS=(Implement* OR Integration* OR deliver* OR sustain* OR Barrier* OR Facilitator*)** | 4,899,500 |

## PsycINFO (368)

| **Search** | **Query** | **Results** |
| --- | --- | --- |
| #4 | Search: **#1 AND #2 AND #3** | 368 |
| #3 | DE "Vocational Rehabilitation" OR DE "Supported Employment" OR DE "Vocational Evaluation" OR DE "Work Adjustment Training" **OR TI(Vocational*[tiab] OR Employment*[tiab] OR "Individual placement and support"[tiab] OR "return to work"[tiab] OR occupation*[tiab]) OR AB(Vocational*[tiab] OR Employment*[tiab] OR "Individual placement and support"[tiab] OR "return to work"[tiab] OR occupation*[tiab]) OR KW(Vocational*[tiab] OR Employment*[tiab] OR "Individual placement and support"[tiab] OR "return to work"[tiab] OR occupation*[tiab])** | 7,986 |
| #2 | (DE "Social Security") **OR DE "Health Care Services" OR DE "Behavioral Health Services" OR DE "Continuum of Care" OR DE "Electronic Health Services" OR DE "Gender Affirming Care" OR DE "Health Care Delivery" OR DE "Health Screening" OR DE "Hospital Programs" OR DE "Long Term Care" OR DE "Mental Health Services" OR DE "Palliative Care" OR DE "Patient Centered Care" OR DE "Primary Health Care" OR DE "Reproductive Health Care" OR TI("health agenc*"[tiab] OR "health service*"[tiab] OR "health staff"[tiab] OR "service staff"[tiab] OR "employment service*"[tiab] OR "insurance service*"[tiab] OR "welfare service*"[tiab] OR "vocational service*"[tiab] OR "Social Insurance*"[tiab] OR "social securit*"[tiab] OR "rehabilitation service*"[tiab] OR "community service*"[tiab] OR Employment-service*[tiab] OR "community mental health*"[tiab]) OR AB("health agenc*"[tiab] OR "health service*"[tiab] OR "health staff"[tiab] OR "service staff"[tiab] OR "employment service*"[tiab] OR "insurance service*"[tiab] OR "welfare service*"[tiab] OR "vocational service*"[tiab] OR "Social Insurance*"[tiab] OR "social securit*"[tiab] OR "rehabilitation service*"[tiab] OR "community service*"[tiab] OR Employment-service*[tiab] OR "community mental health*"[tiab]) OR KW("health agenc*"[tiab] OR "health service*"[tiab] OR "health staff"[tiab] OR "service staff"[tiab] OR "employment service*"[tiab] OR "insurance service*"[tiab] OR "welfare service*"[tiab] OR "vocational service*"[tiab] OR "Social Insurance*"[tiab] OR "social securit*"[tiab] OR "rehabilitation service*"[tiab] OR "community service*"[tiab] OR Employment-service*[tiab] OR "community mental health*"[tiab])** | 227,799 |
| #1 | **DE "Integrated Services" OR DE "Health Care Services" OR DE "Behavioral Health Services" OR DE "Continuum of Care" OR DE "Electronic Health Services" OR DE "Gender Affirming Care" OR DE "Health Care Delivery" OR DE "Health Screening" OR DE "Hospital Programs" OR DE "Long Term Care" OR DE "Mental Health Services" OR DE "Palliative Care" OR DE "Patient Centered Care" OR DE "Primary Health Care" OR DE "Reproductive Health Care" OR DE "Mental Health Programs" OR DE "Crisis Intervention Services" OR DE "Deinstitutionalization" OR DE "Home Visiting Programs" OR DE "Hot Line Services" OR DE "Suicide Prevention Centers" OR DE "Mental Health Services" OR DE "College Mental Health Services" OR DE "Community Mental Health Services" OR DE "Psychological First Aid" OR DE "School Based Mental Health Services" OR DE "Public Health Services" OR DE "Public Health Campaigns" OR DE "Public Service Announcements" OR DE "Social Services" OR DE "Adult Day Care" OR DE "Community Services" OR DE "Elder Care" OR DE "Family Preservation" OR DE "Family Reunification" OR DE "Foster Care" OR DE "Government Programs" OR DE "Independent Living Programs" OR DE "Outreach Programs" OR DE "Protective Services" OR DE "Social Programs" OR DE "Support Groups" OR TI(Implement*[tiab] OR Integration*[tiab] OR deliver*[tiab] OR sustain*[tiab] OR Barrier*[tiab] OR Facilitator*[tiab]) OR AB(Implement*[tiab] OR Integration*[tiab] OR deliver*[tiab] OR sustain*[tiab] OR Barrier*[tiab] OR Facilitator*[tiab]) OR KW(Implement*[tiab] OR Integration*[tiab] OR deliver*[tiab] OR sustain*[tiab] OR Barrier*[tiab] OR Facilitator*[tiab])** | 159,011 |

##

# Final Number of References [17/04/2023]:

| **Database** | **Result** | **After deduplication** |
| --- | --- | --- |
| PubMed | 11,511 | 11,500 |
| Web of Science | 4,821 | 0 |
| PsycINFO | 368 | 283 |
| **Total** | 16,700 | 11,783 |

# Step 2: Training the AI tool ASReview to reorder the articles based on relevance.

The ASReview tool was trained by researcher (YN an AM) with six pre-selected articles (selected by YN) and 25 at random selected articles by ASReview (classified by YN and AM).

| **nr** | **Author + title** | **Selection:** | **Classified:** | **Included based on full text?** |
| --- | --- | --- | --- | --- |
| 1 | 'Moe, C. et al. Implementing individual placement and support (IPS): the experiences of employment specialists in the early implementation phase of IPS in Northern Norway. The IPSNOR study. DOI: 10.1186/s12888-021-03644-x. | Pre-selected | relevant | Yes |
| 2 | Latimer, E. et al. Implementation of supported employment in the context of a national Canadian program: Facilitators, barriers and strategies. DOI: <http://dx.doi.org/10.1037/prj0000355> | Pre-selected | Relevant | No (no b/f mentioned) |
| 3 | Hutchinson, J. et al. Implementing Supported Employment. Lessons from the Making IPS Work Project. DOI: https://doi.org/10.3390/ijerph15071545 | Pre-selected | Relevant | Yes |
| 4 | Wihlman, U. et al. Barriers of inter-organisational integration in vocational rehabilitation. DOI: <https://doi.org/10.5334/ijic.234> | Pre-selected | Relevant | Yes |
| 5 | Menear, M. et al. Organizational analysis of Canadian supported employment programs for people with psychiatric disabilities. DOI: <https://doi.org/10.1016/j.socscimed.2011.02.005> | Pre-selected | Relevant | Yes |
| 6 | Isett, KR. et al. The state policy context of implementation issues for evidence-based practices in mental health. DOI: https://doi.org/10.1176/ps.2007.58.7.914 | Pre-selected | Relevant | Yes |
| 7 | Wang, Q. et al. Exploration on the optimization of occupational injury and employment protection of takeout workers in the context of public health. DOI: 10.3389/fpubh.2023.1115128 | At random | Not relevant (not about VR) | No |
| 8 | Wang, H. et al. Health inequities in COVID-19 vaccination among the elderly: Case of Connecticut. DOI: 10.1016/j.jiph.2021.07.013 | At random | Not relevant (not about VR) | No |
| 9 | Quinn, D. et al. The practice of conscious sedation by Senior Dental Surgeons in the Health Board Dental Service in the Republic of Ireland. PMID: 16830836 | At random | Not relevant (not about VR) | No |
| 10 | Coreil, J. Use of ethnographic research for instrument development in a case-control study of immunization use in Haiti. DOI: 10.1093/ije/18.supplement_2.s33 | At random | Not relevant (not about VR) | No |
| 11 | Glied, S. et al. Child outpatient mental health service use: why doesn't insurance matter? DOI: 10.1002/(sici)1099-176x(199812)1:4<173::aid-mhp23>3.0.co;2-7. | At random | Not relevant (not about VR) | No |
| 12 | Vézina, M. et al. Workplace prevention and promotion strategies. DOI: 10.12927/hcpap..16822. | At random | Not relevant (not about VR) | No |
| 13 | Thomas PA. Preparing nursing students for practice: successful implementation of a clinical practicum in occupational health nursing. PMID: 7626149 | At random | Not relevant (not about VR) | No |
| 14 | Chan, EYL. Et al. Occupational rehabilitation services provided by a community workers health centre in Hong Kong: a case study. PMID: 18198433 | At random | Relevant | No (Intervention was not targeted on people with mental health problems) |
| 15 | Golding-Day, M. et al. Interventions to reduce dependency in bathing in community dwelling older adults: a systematic review. DOI: 10.1186/s13643-017-0586-4. | At random | Not relevant (not about VR) | No |
| 16 | Ahmed, N. et al. Community integration and life satisfaction among individuals with spinal cord injury living in the community after receiving institutional care in Bangladesh. DOI: 10.1080/09638288.2017.1283713. | At random | Not relevant (not about VR) | No |
| 17 | Henneberger, PK, et al. A comparison of work-exacerbated asthma cases from clinical and epidemiological settings. DOI: 10.1155/2013/495767 | At random | Not relevant (not about VR) | No |
| 18 | Neergaard, MA. Et al. Shared care in basic level palliative home care: organizational and interpersonal challenges. DOI: 10.1089/jpm.2010.0036. | At random | Not relevant (not about VR) | No |
| 19 | Hammond, JS. et al. HIV, trauma, and infection control: universal precautions are universally ignored. PMID: 2342138 | At random | Not relevant (not about VR) | No |
| 20 | Rogers, NL. Et al. Consumer perspectives on reasons for unsuccessful VR case closure: An exploratory study. DOI: 10.3233/JVR-2011-0543 | At random | Relevant | No (No implementation described) |
| 21 | Pak, VM. Et al. Risks of phthalate exposure among the general population: implications for occupational health nurses. DOI: 10.1177/216507990705500102 | At random | Not relevant (not about VR) | No |
| 22 | Darawsheh WB. Et al. Awareness and Knowledge about Occupational Therapy in Jordan. DOI: 10.1155/2018/2493584. | At random | Not relevant (not about VR) | No |
| 23 | Mohr DC. Et al. Integrated Employee Occupational Health and Organizational-Level Registered Nurse Outcomes. DOI: 10.1002/(sici)1099-176x(199812)1:4<173::aid-mhp23>3.0.co;2-7 | At random | Not relevant (not about VR) | No |
| 24 | Morgan, MA. Et al. Cancer survivorship: history, quality-of-life issues, and the evolving multidisciplinary approach to implementation of cancer survivorship care plans. DOI: 10.1188/09.ONF.429-436. | At random | Not relevant (not about VR) | No |
| 25 | Laszlo, A. et al. Creating employment opportunities for people with disabilities in healthcare: the Bristol Employment Collaborative. DOI: 10.3233/WOR-2012-1302 | At random | Relevant | No (No implementation described) |
| 26 | Oliffe, JR. et al. Segmenting or Summing the Parts? A Scoping Review of Male Suicide Research in Canada. DOI: 10.1177/07067437211000631. | At random | Not relevant (not about VR) | No |
| 27 | Jimenez, N. et al. Outpatient Rehabilitation for Medicaid-Insured Children Hospitalized With Traumatic Brain Injury. DOI: 10.1542/peds.2015-3500 |  | Not relevant (not about VR) | No |
| 28 | Zachry, AH. Et al. Infant Positioning, Baby Gear Use, and Cranial Asymmetry. DOI: 10.1007/s10995-017-2344-6. |  | Not relevant (not about VR) | No |
| 29 | Jiang, C. et al. A quality improvement initiative to increase Tdap (tetanus, diphtheria, acellular pertussis) vaccination coverage among direct health care providers at a children's hospital. DOI: 10.1016/j.vaccine.2017.11.071. |  | Not relevant (not about VR) | No |
| 30 | Hansson, A. et al. Flawed communications: Health professionals' experience of collaboration in the care of frail elderly patients. DOI: 10.1177/1403494817716001 |  | Not relevant (not about VR) | No |
| 31 | Maurer, F. et al. Effectiveness of CBT on Unemployed Compared to Employed Individuals Suffering from Prevalent Mental Disorders - A Naturalistic Study. DOI: 10.1055/s-0042-124504. |  | Not relevant (not about VR) | No |
