# Supplementary material for: Barriers and facilitators of collaboration during the implementation of vocational rehabilitation interventions: a systematic review
Source: BMC Psychiatry. 2024 Nov 1;24:759. doi: 10.1186/s12888-024-06223-y (PMC11529217; doi:10.1186/s12888-024-06223-y)
Supplement: Supplementary file 3 — Supplementary Material 3: Terms considered to be on collaboration. [file 12888_2024_6223_MOESM3_ESM.docx]

**Appendix 3 – terms considered to be on collaboration**

Building a bridge between

Building trust between

Building stakeholder networks

Collaboration

Collaborative structures

Collaborative protocol

Collaborative working

Community

Communication

Contact

Cooperation

Coordinators

Cross-agency workgroups

Embedding

Human relationship

Integration

Intergratie (NL)

Integrated services

Interagency collaboration

Interagency cooperation

Interdisciplinary team

Inter-organizational integration

Interprofessional communication

Involvement

Leadership

Multi-disciplinary decisions

Negotiations

Network

Partnership

Relationship

Relationship building

Samenwerking (NL)

Sharing

Support

Transmission of information

Teams

Team meetings

Teamwork

Working together as a team
